# Zika virus infection in pregnancy: Establishing a case definition for clinical research on pregnant women with rash in an active transmission setting

**DOI:** 10.1371/journal.pntd.0007763

**Published:** 2019-10-07

**Authors:** Ricardo Arraes de Alencar Ximenes, Demócrito de Barros Miranda-Filho, Elizabeth B. Brickley, Ulisses Ramos Montarroyos, Celina Maria Turchi Martelli, Thalia Velho Barreto de Araújo, Laura C. Rodrigues, Maria de Fatima Pessoa Militão de Albuquerque, Wayner Vieira de Souza, Priscila Mayrelle da Silva Castanha, Rafael F. O. França, Rafael Dhália, Ernesto T. A. Marques, on behalf of the Microcephaly Epidemic Research Group (MERG)

**Affiliations:** 1 Departamento de Medicina Tropical, Universidade Federal de Pernambuco, Recife, PE, Brasil; 2 Departamento de Medicina Interna, Universidade de Pernambuco, Recife, PE, Brasil; 3 Department of Infectious Disease Epidemiology, London School of Hygiene & Tropical Medicine, London, United Kingdom; 4 Instituto de Ciências Biológicas, Universidade de Pernambuco, Recife, PE, Brasil; 5 Instituto Aggeu Magalhães, Fundação Oswaldo Cruz, Recife, PE, Brasil; 6 Departamento de Medicina Social, Universidade Federal de Pernambuco, Recife, PE, Brasil; 7 Department of Infectious Diseases and Microbiology, University of Pittsburgh, Pittsburgh, Pennsylvania, United States of America; 8 Faculdade de Ciências Médicas, Universidade de Pernambuco, Recife, PE, Brasil; University of Queensland, AUSTRALIA

## Abstract

Defining cases of Zika virus (ZIKV) infection is a critical challenge for epidemiological research. Due to ZIKV’s overlapping clinical features and potential immunologic cross-reactivity with other flaviviruses and the current lack of an optimal ZIKV-specific diagnostic assay, varying approaches for identifying ZIKV infections have been employed to date. This paper presents the laboratory results and diagnostic criteria developed by the Microcephaly Epidemic Research Group for defining cases of maternal ZIKV infection in a cohort of pregnant women with rash (N = 694) recruited during the declining 2015–2017 epidemic in northeast Brazil. For this investigation, we tested maternal sera for ZIKV by quantitative reverse transcription polymerase chain reaction (qRT-PCR), Immunoglobulin (Ig) M and IgG3 enzyme-linked immunosorbent assays (ELISAs), and Plaque Reduction Neutralization Test (PRNT_50_). Overall, 23.8% of participants tested positive by qRT-PCR during pregnancy (range of detection: 0–72 days after rash onset). However, the inter-assay concordance was lower than expected. Among women with qRT-PCR-confirmed ZIKV and further testing, only 10.1% had positive IgM tests within 90 days of rash, and only 48.5% had ZIKV-specific PRNT_50_ titers ≥20 within 1 year of rash. Given the complexity of these data, we convened a panel of experts to propose an algorithm for identifying ZIKV infections in pregnancy based on all available lines of evidence. When the diagnostic algorithm was applied to the cohort, 26.9% of participants were classified as having robust evidence of a ZIKV infection during pregnancy, 4.0% as having moderate evidence, 13.3% as having limited evidence of a ZIKV infection but with uncertain timing, and 19.5% as having evidence of an unspecified flavivirus infection before or during pregnancy. Our findings suggest that integrating longitudinal data from nucleic acid and serologic testing may enhance diagnostic sensitivity and underscore the need for an on-going dialogue regarding the optimization of strategies for defining cases of ZIKV in research.

## Author summary

On February 1, 2016, the World Health Organization declared a Public Health Emergency of International Concern following a “cluster of microcephaly cases and other neurological disorders in Brazil” and highlighted the “urgent need” for coordinated international efforts to investigate the relationship between maternal Zika virus (ZIKV) infections and microcephaly. Due to the lack of a standard algorithm, resultant epidemiological investigations have utilized different strategies for defining cases of ZIKV infections in pregnancy. Here, we report the experience of the Microcephaly Epidemic Research Group in Pernambuco, Brazil, in evaluating 694 pregnant women presenting with rash (i.e., a common sign of ZIKV infection) during the 2015–2017 Latin American outbreak. Integrating time-sensitive data from both nucleic acid amplification testing and serologic assays, a panel of experts developed an evidence-graded set of criteria for identifying cases of maternal infection. When applied to the cohort, nearly one-third of the participants were categorized as having robust or moderate evidence of being infected with ZIKV in pregnancy. The classifications described in this investigation will enable scientists to investigate maternal ZIKV infection and estimate the absolute and relative risks of adverse pregnancy outcomes. The results also underscore the importance of on-going efforts to develop robust diagnostic assays for ZIKV.

## Introduction

Defining cases is a universal challenge of epidemiological studies on Zika virus (ZIKV). This problem is exacerbated in regions with co-circulating arthropod-borne viruses (arboviruses) due to overlapping and often mild clinical features [1], the potential for immunologic cross-reactivity with other flaviviruses [2–4], and the current lack of an optimal ZIKV-specific diagnostic assay for diagnosing recent infections [5–7]. As a consequence, different clinical and laboratory criteria have been used to identify ZIKV exposures for the published investigations evaluating pregnancy outcomes after maternal ZIKV infection in Brazil [8], in the French territories of the Americas [9], and in the United States (U.S.) and their territories and freely associated states [10, 11].

The epidemiological case definitions used to define maternal ZIKV infections in recent studies reflect pragmatic considerations (e.g., availability and affordability of relevant diagnostic tests), the recency of sample collections relative to the suspected infections (e.g., timing in returning travelers), and the local epidemiological contexts (e.g., presence or absence of autochthonous transmission, circulation of other flaviviruses). In the investigations by Brasil, *et al*. (2016) [8] and Hoen, *et al*. (2018) [9], which enrolled symptomatic women from settings with active transmission in Brazil and the French territories, it was feasible to collect biological specimens during acute infection, and ZIKV infection in pregnancy was exclusively identified by quantitative reverse transcription polymerase chain reaction (qRT-PCR). In contrast, in the studies by Reynolds, *et al*. (2017) [10] and Shapiro-Mendoza, *et al*. (2017) [11], which were based on the U.S. Zika Pregnancy and Infant Registry, exposure was defined using combination of assays (i.e., qRT-PCR and Plaque Reduction Neutralization Test (PRNT_90_) for ZIKV and immunoglobulin (Ig) M for both ZIKV and DENV) that reflected the varying time windows to sample testing. Similarly, in the investigation by Pomar, *et al*. (2018), which enrolled symptomatic and asymptomatic pregnant women in western French Guiana, ZIKV exposure was laboratory-confirmed by qRT-PCR, IgM and a micro-neutralizing assay; notably, this investigation also included subsequent IgM cord blood testing of the neonates to confirm congenital infection [12].

The Northeast of Brazil, including the state of Pernambuco, was the epicenter of the 2015–2017 microcephaly epidemic associated with ZIKV infection [13]. The initial wave of ZIKV transmission in Pernambuco occurred in the first half of 2015, and a peak of microcephaly cases was observed among neonates born later in the same year (reviewed in [14]). In response to this cluster of congenital abnormalities, the Brazilian Ministry of Health declared a public health emergency [15], and epidemiological investigations into the causal factors were considered to be of paramount importance. To meet this challenge, the Microcephaly Epidemic Research Group (MERG; http://www.cpqam.fiocruz.br/merg/) was formed in late 2015 and rapidly launched two epidemiological investigations: a case-control study of microcephaly, for which the results have been published [16–18], and a cohort study of pregnant women with rash, for which analysis is on-going. In addition to confirming the link between ZIKV and microcephaly [16, 17], the MERG case-control study demonstrated that 85% of pregnant women participating in the Pernambuco-based study had experienced dengue virus (DENV) infections prior to the time of their deliveries [18].

Initiating a large-scale outbreak investigation under financial constraints during a live public health emergency introduced challenging considerations for resource mobilization and prioritization in the clinical studies, and strategic decisions were made by MERG to optimize the effectiveness of the ZIKV testing regimes. This paper presents the laboratory results and the specific diagnostic criteria developed by MERG to define maternal infections in the cohort of pregnant women with rash recruited in Pernambuco State, Brazil, during the declining ZIKV epidemic. Here, we describe MERG’s methods for integrating complex longitudinal data from qRT-PCR, IgM, IgG3, and PRNT_50_ assays in order to establish case definitions for ZIKV infections in pregnancy, discuss the advantages and limitations of this approach, and provide specific recommendations for future prospective cohort studies of ZIKV infections in pregnancy.

## Methods

### Study design and participants

**Fig 1:**
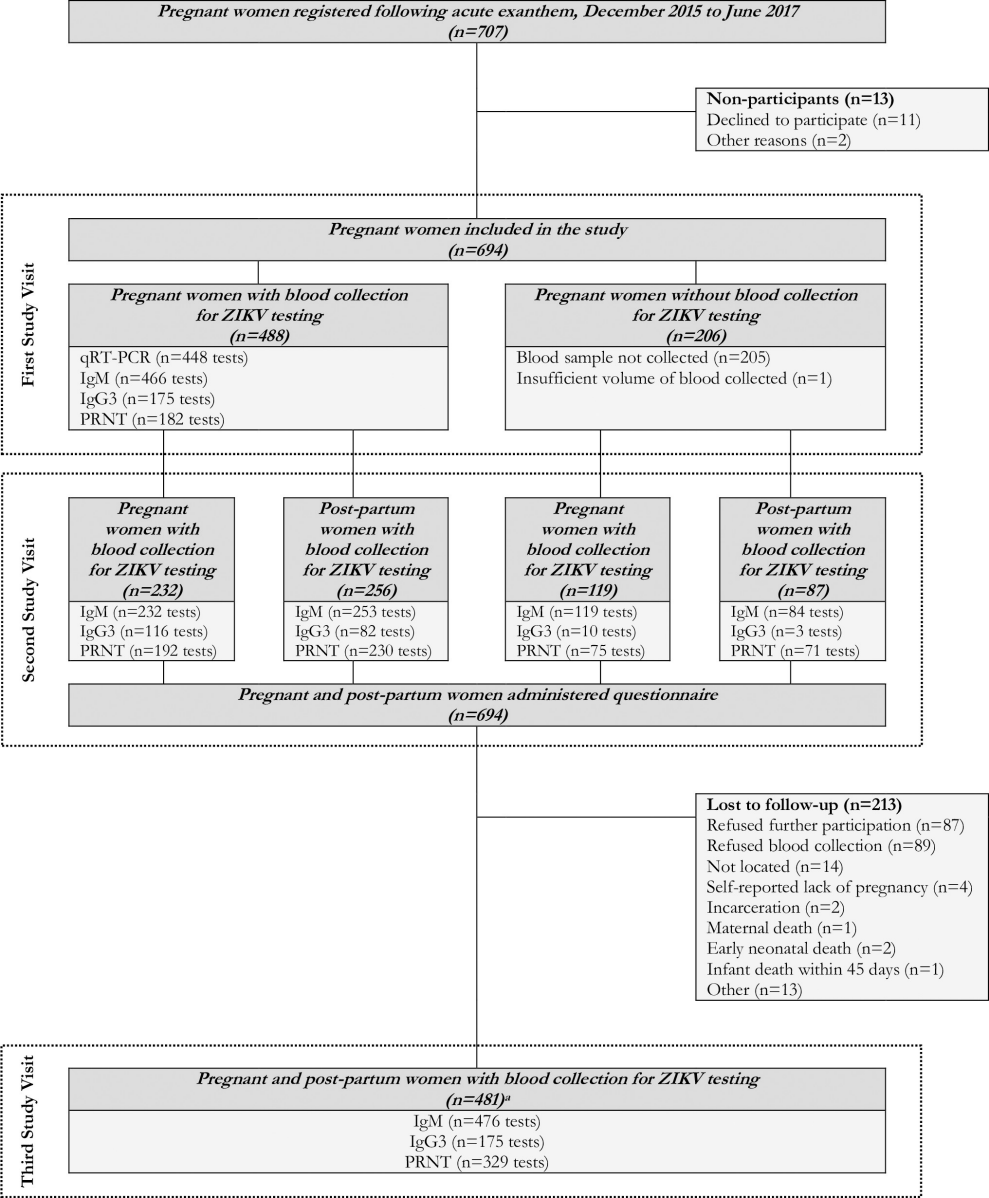
Flow diagram of molecular and serologic testing for ZIKV infection using qRT-PCR, IgM, IgG3, and PRNT_50_ in a cohort of pregnant women with rash in Pernambuco, Brazil.

Following its launch in November 2015, MERG partnered with the Pernambuco State Health Department to develop harmonized protocols to facilitate synergistic activities between research and surveillance. Based on these consultations, the Pernambuco State Health Department introduced a surveillance system for pregnant women presenting with rash (Center for Strategic Information on Health Surveillance in Pernambuco; Cievs/PE) in December 2015. The surveillance program included no restrictions regarding the type of rash. At the time of notification to Cievs/PE −and ideally within five days of rash onset, as recommended in the harmonized protocols− officials from the State Health Secretariat registered women and collected a first blood sample for ZIKV testing (Fig 1). MERG then invited the pregnant women registered in Cievs/PE to participate in a prospective investigation of ZIKV infection in pregnancy; no exclusion criteria were applied. Initial recruitment was limited to the metropolitan region of Recife; however, beginning in April 2016, the catchment area for recruitment was expanded beyond Recife to include additional women with laboratory evidence for ZIKV residing within approximately 120km of the city. MERG-associated fieldworkers collected a second blood sample from the pregnant women (i.e., at least 14 days following initial notification) and administered a detailed questionnaire. In cases of livebirth, a third blood sample was collected after delivery. All blood samples were sent to the Central Laboratory of Public Health (Recife, Pernambuco) where serum samples were separated and stored at -80°C until further diagnostic testing was performed at the Laboratorio de Virologia e Terapia Experimental of the Fundação Oswaldo Cruz (LaViTE-FIOCRUZ, Recife, Pernambuco).

### Laboratory procedures

The molecular and serologic diagnostic assays were performed at LaViTE-Fiocruz and have been subject to external quality assessments within Fiocruz and international reference networks (e.g., see [19]). Maternal sera were tested for the detection of ZIKV genome by one-step qRT-PCR using primers and probes previously described by Lanciotti and colleagues [4]. Specifically, the RNA was extracted automatically from human serum using a QIAmp Viral RNA kit according to the manufacturer’s instructions (Qiagen, Hilden, Germany). The ZIKV qRT-PCR was performed in duplicate in a final volume of 20μL with the GoTaq 1-step RT-qPCR system according to the manufacturer’s instructions (Promega Corporation, Madison, Wisconsin, United States of America). Cycling was performed using the QuantStudio 5 Real-Time PCR System (ThermoFisher Scientific, Waltham, Massachusetts, United States of America). A standard curve for ZIKV RNA copies was prepared from a previously titrated virus stock (range: 10^1^ to 10^6^ PFU/mL). Samples with a Ct value <38 in duplicate wells were considered to be positive for ZIKV. No false positives have been detected in any of the external quality assessments performed to date.

The serologic assays used in this study were selected based on their ability to discern recent infection (i.e., IgM and IgG3) as well as their diagnostic sensitivity and specificity (i.e., PRNT). Samples were screened for the detection of ZIKV-specific IgM antibodies by capture-IgM enzyme-linked immunosorbent assay (ELISA) following a protocol recommended by the United States Centers for Disease Control and Prevention (CDC, Fort Collins, Colorado, United States of America) [20]. Results were calculated as a ratio of the average optical density (OD) value of test sample (P) divided by the average OD value of the negative control (N). Maternal sera were considered ZIKV-equivocal with an IgM ratio >2 and ≤3 and ZIKV-positive with an IgM ratio of >3. Seroconversion by IgM was considered to occur based on two samples, with a switch from negative (i.e., ≤2) to positive status (i.e., >3). Maternal sera were also tested for the detection of ZIKV-specific IgG3 anti-non-structural protein 1 (NS1) antibodies using a novel in-house ELISA, which assessed ZIKV exposure in the past six months, following a protocol previously described [21]. Serum samples, in duplicate, were tested in parallel using NS1 from ZIKV and DENV1-4 as antigens. Assay controls included: ZIKV-positive sera from convalescent patients collected 60 days post-onset of symptoms, dengue-positive pooled sera from early convalescent patients collected 20 to 30 days post-onset of symptoms, and flavivirus-naïve sera (i.e., human IgG-fractionated serum purified from U.S. patients and diluted in IgG-depleted human serum at 50mg/mL). Results were calculated as a ratio by dividing the average OD value of the test sample by the average OD value of the dengue positive recent infection control. Maternal sera were considered ZIKV-positive with an IgG3 ratio of >1.2. ZIKV-specific neutralizing activity was assessed in all available maternal sera by PRNT, following a standardized protocol [22] carried out in Vero cells using a virus strain isolated in the study setting (Recife, Pernambuco, Brazil): ZIKV (BR-PE243/2015) [23]. ZIKV-specific neutralizing antibody titers were estimated using a four-parameter non-linear regression and expressed as the reciprocal dilution needed to achieve a 50% reduction in plaque counts (PRNT_50_). Maternal sera were considered ZIKV-non-negative with PRNT_50_ titers ≥20, equivocal with PRNT_50_ titers ≥20 and <100, and ZIKV-positive with PRNT_50_ titers ≥100. Seroconversion was considered to occur with four-fold rises in PRNT_50_ titers or with a switch from negative (i.e., <20) to non-negative status (i.e., ≥20). Subsamples of maternal sera underwent further testing for other potential infections with TORCH agents (toxoplasmosis, parvovirus B19, rubella, cytomegalovirus, herpes simplex virus) and other arboviruses (DENV and Chikungunya virus).

### Expert panel

A panel of experts was assembled to review the evidence and establish a case definition for the cohort of pregnant women with rash. The experts included three virologists, one infectious disease specialist, and one epidemiologist who were all actively engaged in the rapid response to the 2015–2016 public health emergency in Brazil. The panel reviewed all maternal lab results from qRT-PCR, IgM, IgG3, and PRNT_50_ testing in relation to the dates of the rash and the pregnancy. Specific testing regimes varied across participating women, such that not all tests were used for all women during each study visit. Because of this variability, the panel appraised each woman’s test results individually. For each participant, the expert panel first considered the qRT-PCR results in relation to the time since rash. They then examined serially evaluated samples for evidence of seroconversion by either IgM or PRNT. Next, they considered the concordance of the IgM and IgG3 results in relation to the PRNT results (i.e., evaluated in terms of both titer and timing of testing in relation to the end of pregnancy). Finally, they considered the individual serologic test results in the absence of any confirmatory evidence. Based on this detailed evaluation, the panel established by consensus the specific set of rules described in this manuscript for defining cases of ZIKV infection in pregnancy and classified women according to the evidence-graded criteria.

### Ethics statement

The study was approved by the Ethical Committee of the Instituto Aggeu Magalhães (53240816.4.0000.5190) and was conducted in accordance with the Declaration of Helsinki, the International Conference on Harmonisation guideline for Good Clinical Practice, and the codes and regulations of Brazil regarding research on human subjects. Pregnant women provided a written informed consent prior to participating in the study.

## Results

In coordination with this investigation, the Pernambuco State Health Department registered 707 pregnant women between December 2015 and June 2017 following a reported exanthem (Fig 1). 694 women were recruited for further follow-up. Blood samples were collected during the baseline visit from 70.3% (N = 488/694) of women, with a median time from symptom onset to first blood collection of 3 (interquartile range, IQR: 1 to 6) days. During the second study visit, fieldworkers from MERG interviewed and collected blood samples from all 694 women, with a median time to blood collection of 87 (IQR: 56 to 130) days. Second study visits occurred prior to delivery for 50.6% (N = 351/694) of women. During the third study visit, fieldworkers collected blood samples from 69.3% (N = 481/694) of the study sample, with a median time to blood collection of 263.5 (IQR: 154.5 to 417.5) days. 98.8% (N = 475/481) of third study visits occurred after the pregnancy ended. Notably, 87 women completed their pregnancies prior to the time of blood sample collection.

### Diagnostic testing

**Fig 2:**
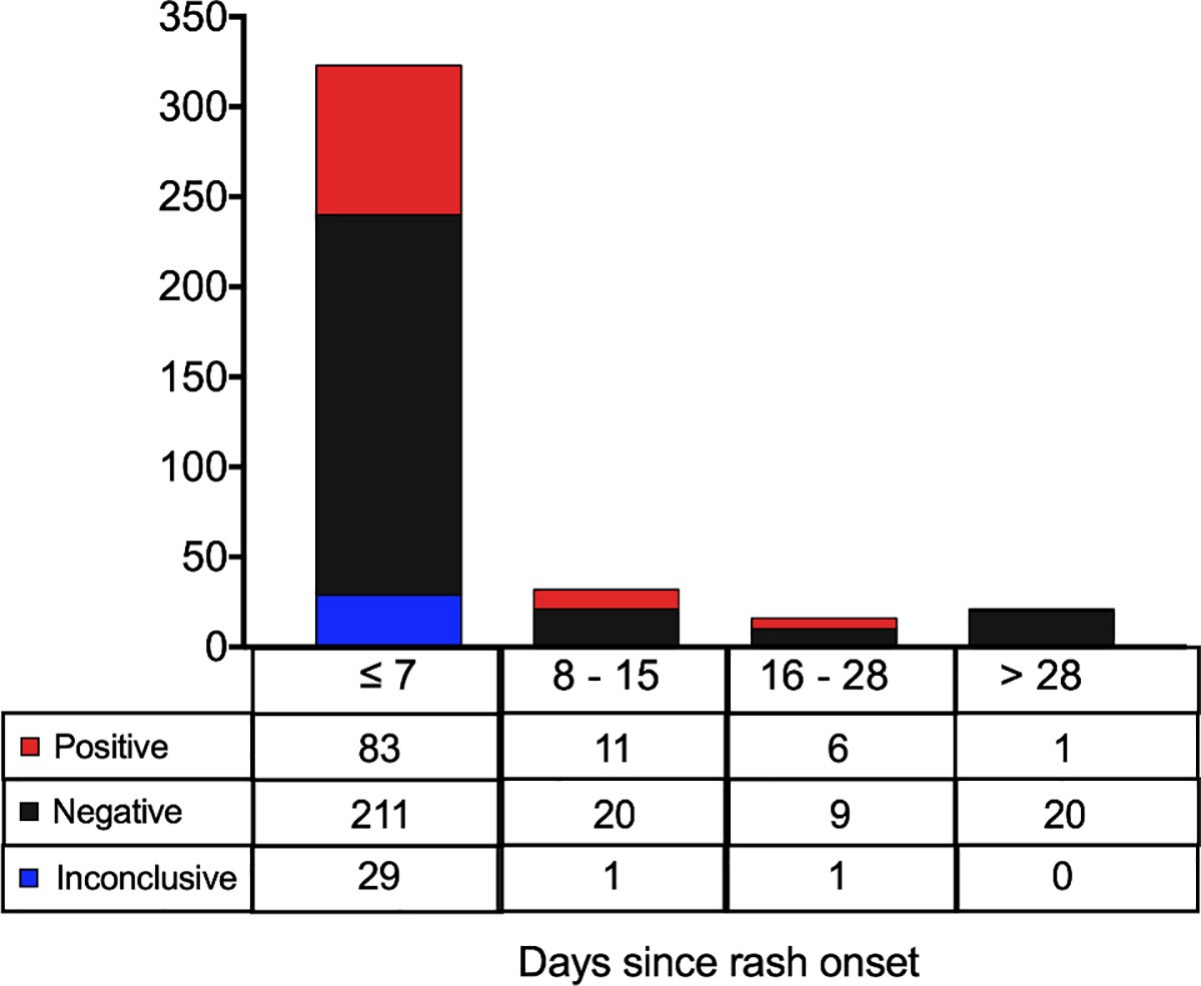
ZIKV-specific qRT-PCR results in relation to time since rash onset.

ZIKV exposure status was evaluated in a total of 1663 serum samples collected from 364 (52.4%) women tested at 3 time points, 240 (34.6%) tested at 2 time points, and 90 (13.0%) tested at 1 time point. Of the 1663 samples, 448 (26.9%) were evaluated by qRT-PCR, 1630 (98.0%) by IgM, 561 (33.7%) by IgG3, and 1079 (64.9%) by PRNT_50_. Of the 448 women whose samples from the first study visit were tested by qRT-PCR, 127 (28.3%) were positive. Whereas 83 (82.1%) of the 101 qRT-PCR-positives with known timing were detected within 7 days of rash onset, viral RNA was also detected in 19 (18.8%) samples collected later than one week after rash onset (Fig 2). The latest qRT-PCR-positive sample was collected 72 days after rash onset. Although fewer samples were collected at the later time points, the likelihood of detecting viral RNA remained constant at approximately 30% for the first 28 days after the appearance of the rash. Strikingly, in 73 of the 127 (57.5%) women who tested positive by qRT-PCR, there was no confirmatory serologic evidence of infection.

**Fig 3:**
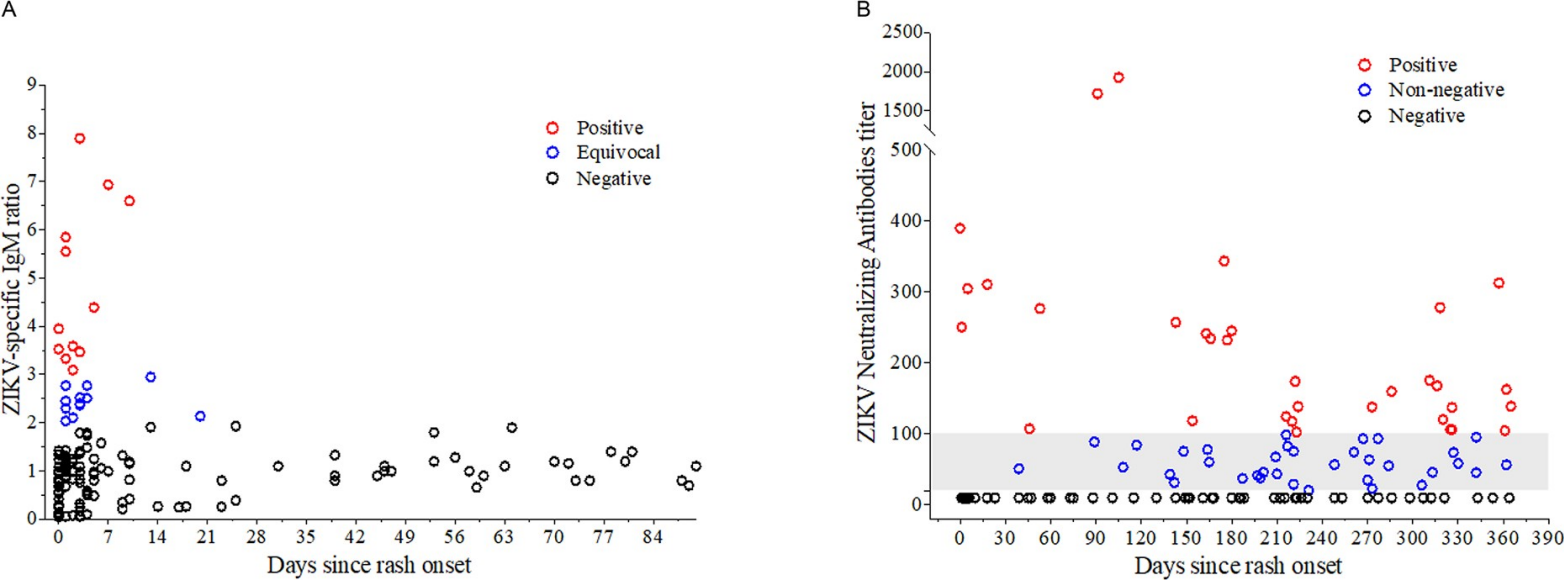
Among women with qRT-PCR-confirmed ZIKV, (A) ZIKV-specific IgM results within 90 days of rash onset and (B) ZIKV-specific PRNT_50_ results within 1 year of rash onset.

Of the 694 women whose samples were tested by IgM, 71 (10.2%) were positive during at least one time point. Similarly, of the 240 women whose samples were tested by IgG3, 20 (8.3%) were positive during at least one time point. Among the 105 women with qRT-PCR-confirmed ZIKV and IgM testing within 90 days of rash, only 11 (10.5%) were observed to have positive IgM tests (Fig 3). Negative test results by IgM were observed for samples collected at the same time as qRT-PCR testing as well as among those collected later. Of note, positive IgM results were observed more than 90 days after rash onset in 5 women with qRT-PCR-confirmed ZIKV at baseline. Among the 21 women with qRT-PCR-confirmed ZIKV and IgG3 testing for ZIKV, only 1 (4.8%) was positive. Of the 581 women evaluated by PRNT_50_, 324 (55.8%) had ZIKV-specific PRNT_50_ titers ≥ 20 during at least one time point, with 312 (96.3%) non-negative women exhibiting titers ≥ 100 in at least one time point. Among the 103 women with qRT-PCR-confirmed ZIKV and PRNT_50_ performed within one year of rash, 50 (48.5%) were observed to have ZIKV-specific PRNT_50_ titers ≥20 (Fig 3).

A subsample of 126 women who were qRT-PCR positive for ZIKV underwent further testing for previous DENV infection (i.e., based on IgG antibodies). Within this group, 119 were DENV-positive, 4 were DENV-negative, and 3 were inconclusive. Among the 93 women who were qRT-PCR positive for ZIKV, IgG positive for DENV, and tested for ZIKV IgM within 90 days of the start of rash, 10 (10.8%) were IgM positive, 73 (78.5%) were negative, and 10 (10.7%) were inconclusive. Among the 99 women who were qRT-PCR positive for ZIKV, IgG positive for DENV, and tested by PRNT_50_ within one year of the start of rash, 48 (48.5%) were PRNT_50_ positive and 51 (51.5%) were PRNT_50_ negative. The only woman who was qRT-PCR positive for ZIKV, IgG negative for DENV, and tested by PRNT_50_ within one year of the start of rash was PRNT_50_ negative.

### Evidence of recent ZIKV infection

**Fig 4:**
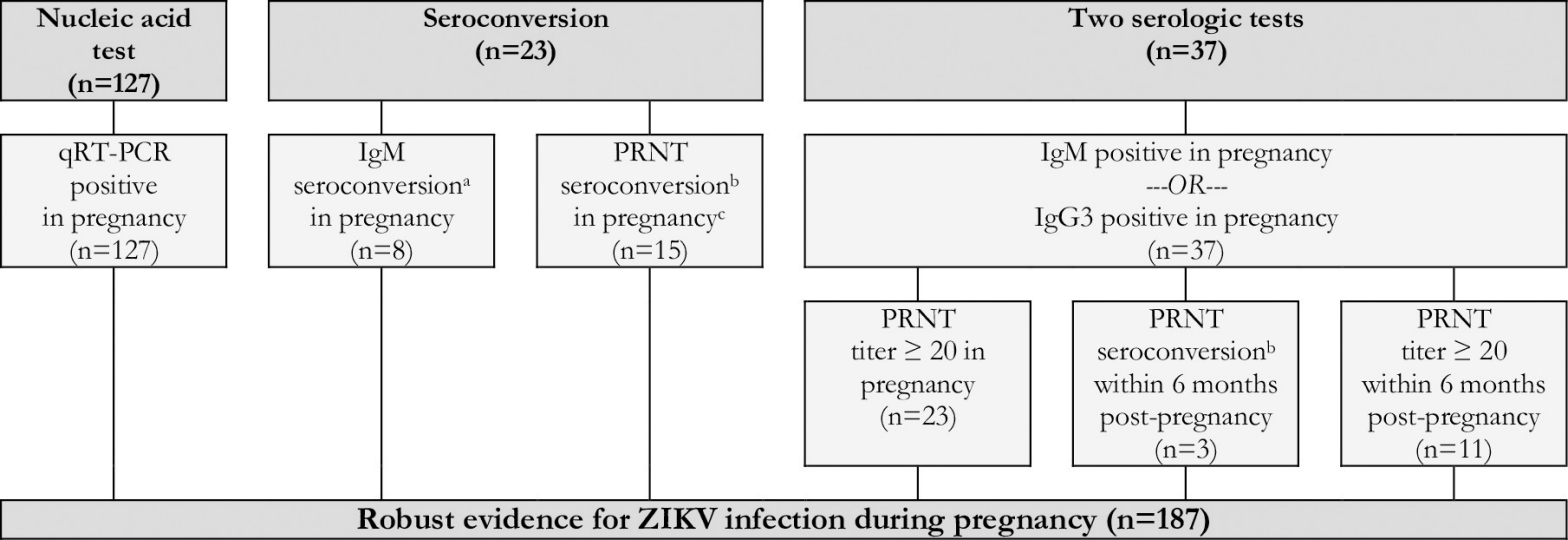
Robust evidence for ZIKV infection in pregnancy based on the results of qRT-PCR, IgM, IgG3, and PRNT_50_ in a cohort of pregnant women with rash in Pernambuco, Brazil.

**Fig 5:**
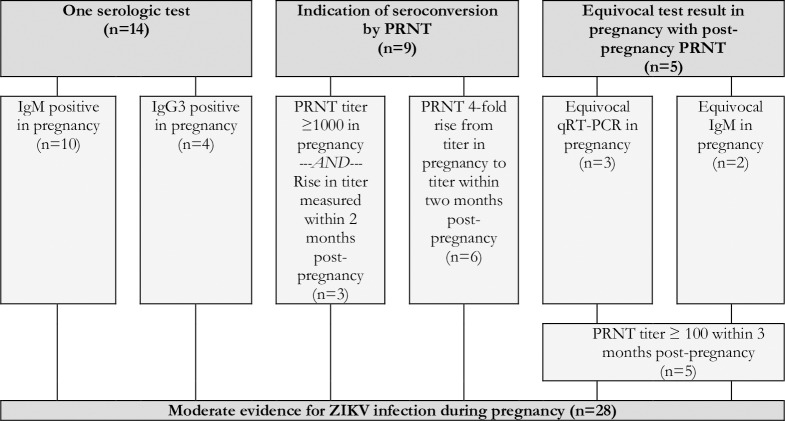
Moderate evidence for ZIKV infection in pregnancy based on the results of IgM, IgG3, and PRNT_50_ in a cohort of pregnant women with rash in Pernambuco, Brazil.

**Fig 6:**
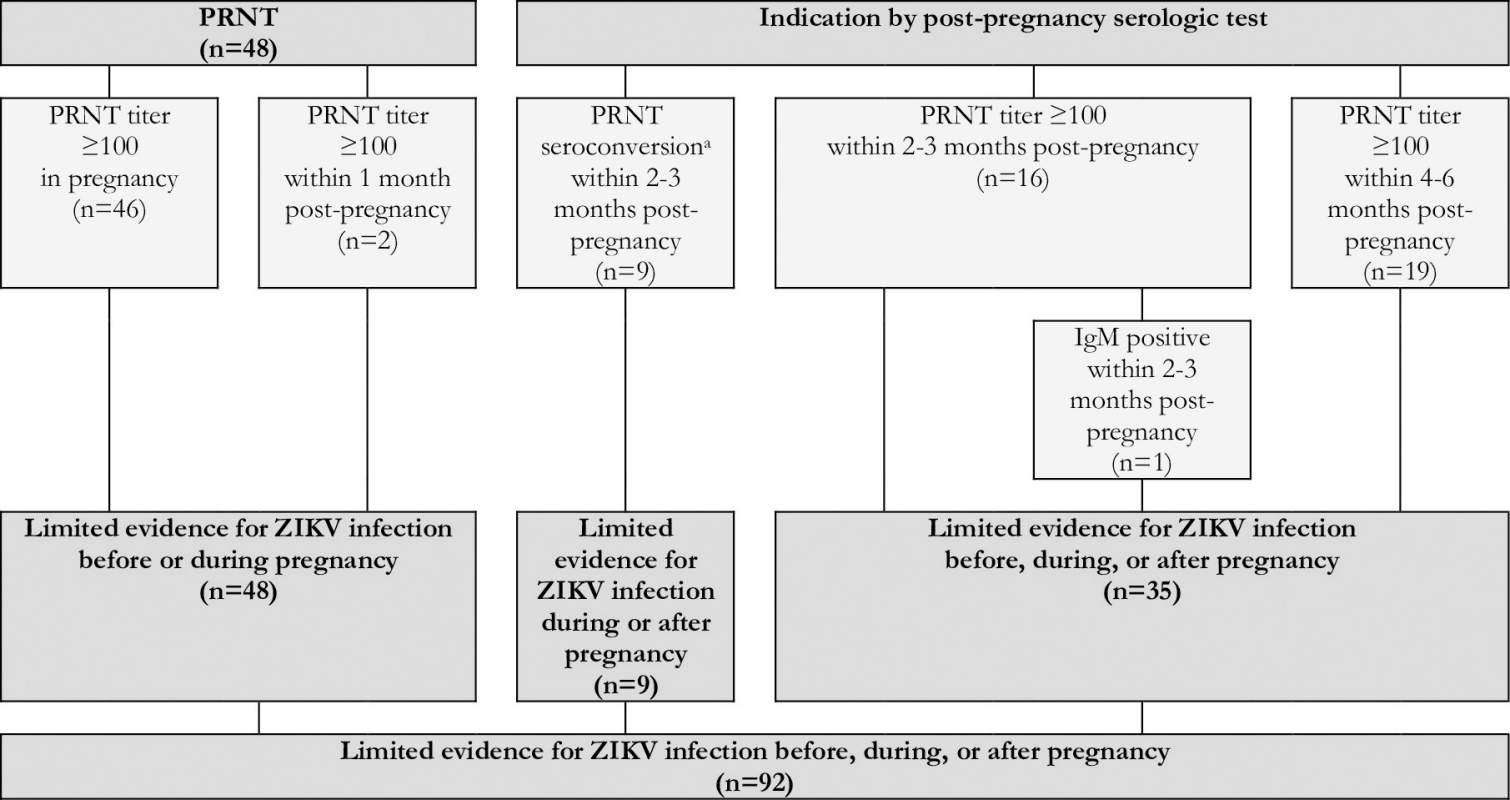
Limited evidence for a ZIKV infection before, during, or after pregnancy based on the results of IgM and PRNT_50_ in a cohort of pregnant women with rash in Pernambuco, Brazil.

**Fig 7:**
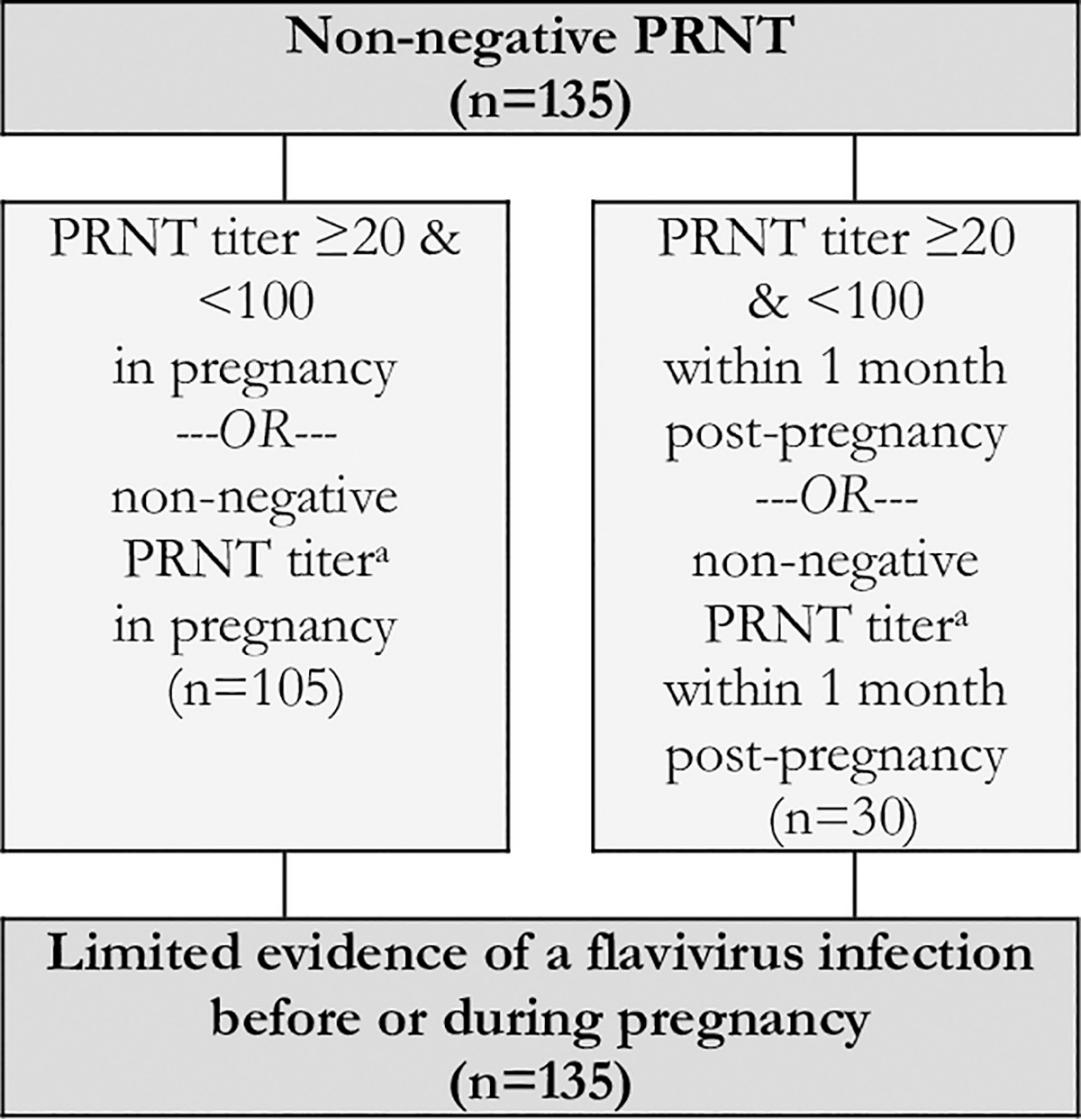
Limited evidence for an unspecified flavivirus infection before or during pregnancy based on the results of PRNT_50_ in a cohort of pregnant women with rash in Pernambuco, Brazil.

**Fig 8:**
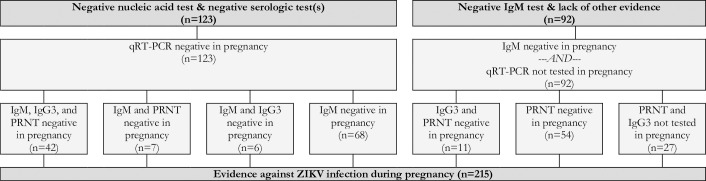
Evidence against a ZIKV infection in pregnancy based on the results of qRT-PCR, IgM, IgG3, and PRNT_50_ in a cohort of pregnant women with rash in Pernambuco, Brazil.

Reflecting the varying degrees of confidence in the specific laboratory assays, the timing of testing in relation to the dates of the rash and the pregnancy, and the availability of confirmatory test results, the expert panel developed and applied a set of definitional criteria to the dataset. Cases were defined as having robust evidence of maternal ZIKV infection if they had a positive nucleic acid amplification test, seroconversion, or at least two positive serologic tests in pregnancy of if they had one positive serologic tests (i.e., IgM or IgG3) in pregnancy paired with a non-negative PRNT_50_ within six months post-pregnancy (Fig 4). Cases were defined as having moderate evidence of maternal ZIKV infection if they had only one positive serologic test (i.e., IgM or IgG3) in pregnancy, an indication of seroconversion by PRNT_50_ during pregnancy (i.e., either a PRNT_50_ titer ≥1000 in pregnancy paired with a rise within 2 months post-pregnancy or 4-fold rise in PRNT_50_ titer from pregnancy to within 2 months post-pregnancy), or an equivocal PRNT_50_ test result in pregnancy paired with a positive PRNT_50_ within three months post-pregnancy (Fig 5). Cases were defined as having limited evidence of ZIKV infection but with uncertain timing relative to the pregnancy if they had a positive PRNT_50_ in pregnancy or within 6 months post-pregnancy or an indication of PRNT_50_ seroconversion during the 2 to 3 months post-pregnancy (Fig 6). Cases were defined as having limited evidence of a flavivirus before or during pregnancy if they had a PRNT_50_ titer between 20 and 100 or a non-negative result (i.e., unspecified titer ≥20) in pregnancy or within 1 month post-pregnancy (Fig 7). Finally, cases were considered to have evidence against ZIKV infection in pregnancy if all tests performed in pregnancy were negative (Fig 8).

Based on these criteria, 187 (26.9%) women had robust evidence of ZIKV infection in pregnancy (Fig 4). Within this group, 127 (67.9%) were qRT-PCR positive for ZIKV. An additional 23 (12.3%) women were observed to seroconvert in pregnancy, based on IgM test results in 8 cases and PRNT_50_ in 15 cases. A further 37 (19.7%) had a positive ZIKV-specific IgM or IgG3 ELISA test result coupled with a PRNT_50_ positive or seroconversion within 6 months post-pregnancy.

A total of 28 (4.0%) women had moderate evidence of ZIKV infection in pregnancy (Fig 5). Within this group, 14 (50.0%) had a positive result for ZIKV-specific IgM or ZIKV-specific IgG3 in pregnancy. An additional 9 (32.1%) had evidence consistent with seroconversion as indicated by PRNT_50_ titers ≥ 1000 with a rising trend or a 4-fold PRNT_50_ titer rise, comparing one sample collected during pregnancy with a second sample collected within 2 months post-pregnancy. A further 5 (17.9%) were considered to have moderate evidence when PCR or ZIKV-specific IgM results were equivocal during pregnancy and there was a positive PRNT_50_ for ZIKV in the three months following pregnancy.

A limited evidence outcome was reached for the 92 cases (13.3%) where a positive indicator of infection was observed, but the timing of the infection in relation to the pregnancy could not be firmly established (Fig 6). Within this group, 48 (52.2%) had a positive PRNT_50_ result during pregnancy or within 1 month post-pregnancy. An additional 9 (9.8%) had a late PRNT_50_ seroconversion, with a negative PRNT_50_ result in pregnancy and a positive PRNT_50_ between 2 and 3 months post-pregnancy. The final 35 (38.0%) had a positive PRNT_50_ observed in the 2 to 6 months post-pregnancy, and it was therefore uncertain whether the ZIKV infection occurred before, during, or after the pregnancy.

Recognizing the potential for cross-reactivity in the immune responses to dengue and Zika viruses, 135 (19.5%) women with equivocal PRNT_50_ results were classified as having limited evidence of an unspecified flavivirus infection in pregnancy (Fig 7). Of these, 105 (77.8%) were detected during pregnancy with the remaining 30 (22.2%) measured within 1 month post-pregnancy.

Overall, 215 (31.0%) women had evidence against ZIKV infection in pregnancy (Fig 8). Although 188 (87.4%) of this group had at least two negative tests in pregnancy, we note that 27 (12.6%) women had evidence against a ZIKV infection in pregnancy by IgM testing alone. Finally, in the 37 (5.3% of 694) women from whom blood could not be collected prior to delivery and for whom there was no post-pregnancy evidence of ZIKV infection, there was limited evidence against ZIKV infection in pregnancy.

## Discussion

Using sera collected around the time of pregnancy from 694 women residing in a region of Northeast Brazil with circulation of Zika and other arboviruses, we used molecular and serologic assays to exhaustively test for ZIKV infections in pregnancy. Based on these test results, a panel of experts developed a set of criteria for defining cases of ZIKV infection during pregnancy for epidemiological research in regions with active flavivirus transmission. These criteria incorporated data from qRT-PCR, IgM, IgG3, and PRNT_50_ assays and aimed to account for heterogeneity in the timing of sample collection relative to a given woman’s exanthem and pregnancy. When applied to the cohort, 26.9% of participants were classified as having robust evidence, 4.0% as having moderate evidence, 13.3% as having evidence of a prior ZIKV infection but with uncertain timing relative to the pregnancy, 19.5% as having evidence of a prior unspecified flavivirus infection, and 31.0% as having no evidence of a ZIKV infection in pregnancy.

The case definition described here contrasts the diagnostic strategies previously reported by Brasil, *et al*. (2016) and Hoen, *et al*. (2018) in two key domains [8, 9]. First, MERG’s definition is hierarchical and provides scope for women to be classified as having moderate to limited evidence of a ZIKV infection. By allowing for there to be a gradation of evidence, this methodology aimed to facilitate sensitivity analyses, to mitigate type two error, and to reduce the potential for biased or attenuated effect estimates that could arise from misclassifications across exposure groups. Second, this case definition relied on qRT-PCR, but also gave weight to the results of serologic tests, in an approach that shares similarities with that described in the CDC guidelines for clinical diagnosis [24]. A key advantage of this method is that it allows for “catch-up” testing of women whose first study visit fell beyond the window of reliable detection for qRT-PCR. In contrast to the U.S. Zika Pregnancy and Infant Registry investigations that rely on a “snapshot” sampling strategy [10, 11], the current method utilizes findings from two or more time points.

Further, as recent studies have demonstrated that approximately 85% of pregnant women in the catchment area have laboratory evidence of prior dengue infection [17, 18], adaptations were made to the CDC guidelines for the interpretation of PRNT results (i.e., PRNT_50_ > 100 versus PRNT_90_ > 20) in order to optimize testing for the specific study context [21, 24].

The results of the laboratory testing add to the evidence base on the diagnosis of ZIKV infections in pregnancy. Specifically, the qRT-PCR results provide important new evidence for practitioners that there may be value in testing pregnant women by molecular methods for up to 28 days after symptom onset. In these data, we observed a similar percentage of positive samples collected in week 1 (26%), week 2 (34%), or weeks 3–4 (38%) after rash (Fig 2). Our findings also indicate that a minority of qRT-PCR-positive pregnant women were observed to have positive results when re-tested by IgM and PRNT_50_ assays at appropriate time intervals during follow-up. Indeed, 73 of the qRT-PCR-positive pregnant women would have been misclassified as ZIKV-negative if we had instead relied exclusively on the serologic test results.

The discordance between the test results is of high public health significance. This finding underscores the importance of rapidly collecting samples for diagnostic testing from symptomatic pregnant women during periods of active ZIKV transmission. Our observation also highlights the potential challenges the public health community faces in correctly diagnosing women without symptoms for whom the timing of infection, and therefore the anticipated persistence of viremia, may be less certain. Our findings regarding the low IgM sensitivity aligns with earlier reports of travelers with previous DENV experience [2] and among the mothers of neonates born with microcephaly during the ZIKV outbreak in northeast Brazil [18]. Together, these results suggest IgM may be inappropriate as a first-line testing method in regions with substantial prior flavivirus transmission. The findings from the PRNT_50_ assays also raise questions about the kinetics of the ZIKV neutralizing antibody responses during pregnancy. Consistent with previous findings from Recife that demonstrated that less than 70% of women who delivered neonates with microcephaly had detectable ZIKV-specific neutralizing antibodies at the time of delivery, our study finds that fewer than 50% of the mothers with qRT-PCR-confirmed ZIKV were found to test positive for ZIKV by PRNT_50_. Together, these results suggest further research is needed to define the utility of PRNT_50_ as a confirmatory testing measure for maternal ZIKV infections.

The strengths of this study warrant consideration. As the cohort investigation was a collaboration between the Pernambuco State Health Secretariat and MERG, the surveillance-based initial sampling leveraged existing local health infrastructure, thereby enabling (i) a real-time response to epidemic conditions with first blood draws occurring at a median of 3 days after rash, (ii) a recruitment strategy with high geographic coverage including in economically deprived communities, and (iii) a higher total number of women invited to participate. Further, as there was a pre-established reference laboratory at LaViTE-FIOCRUZ (i.e., operating since before the beginning of the ZIKV outbreak) with specific expertise in flavivirus diagnostics and ready access to ZIKV-specific immunoassays and primers, there was a uniquely high coverage rate for ZIKV testing and also an opportunity for incorporating complex methods, such as PRNT_50_, and novel approaches, such as IgG3 testing, into the diagnostic criteria [25]. Finally, by incorporating a repeated sampling strategy and multiple lines of evidence, the current approach has been designed to increase the diagnostic sensitivity and to allow for a meaningful and exhaustively tested control group with evidence against ZIKV infection in pregnancy.

On the other hand, the approach described in this study also had important limitations. One limitation is that not all ZIKV tests were available or appropriate for all women at each of the study visits (e.g., qRT-PCR was used almost exclusively near the time of acute rash). Similarly, only a small number of women were tested for DENV IgG, which limits our ability to distinguish between women with and without a previous DENV infection. Although current evidence suggests prior DENV exposure can influence the antibody response to ZIKV [2], we note that approximately 95% of the women tested for DENV IgG had a positive test result, indicating a high degree of homogeneity with respect to prior flavivirus experience across the cohort. These results are consistent with previous publications showing a high circulation of DENV in this area [18, 26]. Another related limitation of this study is the generalizability of the proposed diagnostic algorithm. By design, the criteria set by MERG apply specifically to pregnant women recruited due to exanthem who reside in active arboviral transmission settings and would need to be adapted for the identification of asymptomatic ZIKV infections in pregnancy and other epidemiologic settings. Further, the complexity and costs associated with the described approach limit the utility of these criteria in the clinical (i.e., as opposed to research) context, particularly in resource-limited settings or in the context of an outbreak. Although the MERG algorithm for defining cases of ZIKV infection in pregnancy may not be directly applicable to other study settings, these criteria set a precedent for the transparent reporting of ZIKV case identification.

In addition, several lessons learned from this investigation are important for epidemic preparedness and the planning of future investigations of ZIKV in pregnancy. Our first recommendation is that future prospective cohort studies investigating ZIKV infections in pregnancies should incorporate both nucleic acid and serologic testing platforms for defining cases. qRT-PCR testing is highly specific and thereby mitigates the risk of misclassification that can arise due to immunologic cross-reactivity in serologic tests. Because the window for detecting ZIKV RNA in maternal sera is narrow, a positive result therefore provides an important indicator of the potential gestational age of infection—a factor that may be related to the risk of fetal abnormalities [8]. Despite these advantages, our findings indicate that relying exclusively on nucleic acid amplification testing of maternal sera may substantially underestimate the proportion of the cohort exposed. In this study, we observed that 127 women (18.3%) tested positive by qRT-PCR, while an additional 98 (14.1%) were identified as having moderate-to-robust evidence of ZIKV infection when immunologic metrics were considered.

A second recommendation is that women should be tested, if possible, prior to conception and at more than one time during pregnancy. One advantage is that repeated testing (e.g., with IgM across each trimester in regions without previous circulation of flaviviruses) would enable investigators to detect cases among asymptomatic as well as symptomatic women. This will be important in the future as the existing evidence base suggests there may be similar risks of adverse fetal outcomes arising from symptomatic and asymptomatic maternal ZIKV infections [27]. Another advantage is that repeated testing with PRNT would make it feasible, in regions with previous circulation of ZIKV, to establish seroconversion and therefore determine whether positive results indicate an infection occurring during pregnancy or previously. Although the persistence of ZIKV PRNT positivity remains uncertain, evidence from yellow fever vaccination among travelers suggests that flavivirus-specific neutralizing antibodies may remain detectable in serum for more than ten years [28]. A final advantage is that repeated testing is likely to improve the negative predictive value of diagnostic regimes by increasing the likelihood that samples are tested within assay-appropriate windows of detection. In our study, integrating data from multiple tests and time points enabled us to address temporal sensitivity concerns in order to gather evidence against infection and meaningfully define an unexposed group.

Our third recommendation is that future investigations should consider testing multiple body fluids. Although maternal sera samples were the only testing media collected during the acute outbreak in Pernambuco state, recent case reports indicate that ZIKV may be detected over a longer duration in other fluids including urine, saliva, vaginal secretions, and whole blood [2, 29, 30]. For studies with a primary outcome of Congenital Zika Syndrome, it may also be advantageous to consider testing neonatal samples (e.g., as described in [12]) in order to establish congenital infection more directly.

Our final recommendation is that the development and optimization of diagnostic tools for identifying ZIKV infections must be a priority for epidemic preparedness. Similar to previous reports [2], we observed in this study that a minority of women with qRT-PCR-confirmed ZIKV were also identified as positive by IgM and PRNT_50_ assays. As a first step, it will be vitally important to prioritize studies investigating the persistence of viral replication across body domains and the kinetics of the immune response to ZIKV, especially in vulnerable populations including pregnant women and neonates. Furthermore, due to potential immunologic cross-reactivity, it will be valuable to validate new diagnostic tools among both populations residing in flavivirus-endemic settings and immunologically naïve travelers.

In conclusion, this study provides a model of how complex, longitudinal nucleic acid and serologic test results can be integrated for defining cases of ZIKV infection among pregnant women presenting with rash. The classifications described in this investigation will enable scientists to estimate the absolute and relative risks of adverse pregnancy outcomes associated with maternal ZIKV infections and serve as a model of transparent reporting of case definitions for future epidemiologic investigations conducted during outbreaks. As our understanding of the pathophysiology of ZIKV infection and of the fundamental kinetics of the ZIKV-specific immune response grows and, as new diagnostic tools are introduced, we recognize that there is a need for an on-going dialogue regarding the optimization of strategies for defining cases in epidemiological research.
